# What do cancer patients experience of the simultaneous care clinic? Results of a cross‐sectional study on patient care satisfaction

**DOI:** 10.1002/cam4.7000

**Published:** 2024-02-24

**Authors:** Antonella Galiano, Alessandra Feltrin, Ardi Pambuku, Leda Lo Mauro, Chiara De Toni, Sabina Murgioni, Caterina Soldà, Marco Maruzzo, Francesca Bergamo, Antonella Brunello, Vittorina Zagonel

**Affiliations:** ^1^ Department of Oncology, Oncology Unit 1 Veneto Institute of Oncology IOV‐IRCCS Padua Italy; ^2^ Hospital Psychology Veneto Institute of Oncology IOV‐IRCCS Padua Italy; ^3^ Pain Therapy and Palliative Care Unit Veneto Institute of Oncology IOV‐IRCCS Padua Italy; ^4^ Clinical Nutrition Unit, Veneto Institute of Oncology IOV‐IRCCS Padua Italy

**Keywords:** cancer patients, communication, embedded early palliative care, patient satisfaction, simultaneous care

## Abstract

**Background:**

Veneto Institute of Oncology has activated a simultaneous care outpatient clinic (SCOC) in which cancer patients with advanced‐stage cancer are evaluated by oncologist and palliative care specialists. This cross‐sectional study investigated patients' perceptions of the quality of this service.

**Materials and Methods:**

An ad‐hoc self‐administered questionnaire, developed by SCOC team, was used to assess the satisfaction of patients admitted at SCOC consultation. The questionnaire, in addition to the socio‐demographic questions, contains eight questions with the Likert scale: time dedicated, feel listened to, feel understood, feel free to speak openly and to express doubts and concerns, feeling about information and indication received, level of empathy of health care and quality of the relationship, level of professional/quality of performance and utility of consultation, and one open‐ended question. The questionnaire has been proposed to all 174 consecutively admitted patients at SCOC.

**Results:**

One hundred and sixty‐two patients filled in the questionnaire: 66.7% were male, median age was 71 years, 88.3% had metastatic disease. The time dedicated to SCOC consultation was judged more than adequate (55%) or adequate (35%) by 90% of subjects. Patients completely satisfied about being listened to were 92.5%, with 80.9% being completely satisfied with understanding of their issues and 92% with the freedom to speak and express doubts. Usefulness of the SCOC was rated as excellent by 40% and good by 54.4% of patients. No statistically significant differences were observed in the responses to the questions by gender, age (< or ≥70 years old) and type of tumor.

**Conclusion:**

Our study shows high levels of satisfactions after SCOC consultation in advanced cancer subjects. Patients' feedback confirmed that SCOC model was effective in helping them during their treatment journey and decision at the end of life. This study encouraged us to enhance our practice of SCOC consultation.

**Implications for Practice:**

A joint evaluation of patients living with cancer by oncologist and palliative care team (SCOC‐embedded model), has shown to enhance patients' experience/satisfaction with care‐such as listening, understanding, receiving information, symptom control, and decision about future, independently of age, gender, and kind of tumor.

## 1 INTRODUCTION

Early palliative care (EPC) improves quality of life and care satisfaction of patients with advanced‐stage cancer.^1^, ^2^, ^3^ There is no unique model of EPC delivery that applies to all settings,^4^ albeit a close integration among oncologists and interdisciplinary palliative care team is recommended.^2^, ^5^ In particular, a stand‐alone clinic or an embedded clinic are the two principal models for outpatient EPC.^6^ Veneto Institute of Oncology (IOV) is a Comprehensive Cancer Centre in Italy in which in 2014 a simultaneous care outpatient clinic (SCOC) was activated. In the SCOC, patients with advanced cancer are evaluated by an interdisciplinary team composed of an oncologist, a palliative care specialist, a clinical nutrition specialist, a psycho‐oncologist and a nurse navigator, establishing an EPC approach as suggested by clinical and scientific evidence.^5^, ^7^ This fully embedded and innovative organizational model, in which the oncologist and the palliative care team share the SCOC, allows for a direct interaction between specialists, and at the same time intercept cancer patients with advanced‐stage cancer who need global care. As previously reported, patients are referred to the SCOC by oncologists, who fill out a form that contains a score based on some of the patient's parameters: symptoms, Karnofsky Performance status, estimated survival, availability of cancer‐directed treatment with impact on survival, expected toxicity from anticancer therapy, and presence of social issues.^8^, ^9^ Access of patients to the SCOC is prioritized based on the final score. The opportunity of SCOC visits is encouraged by Oncologists, who inform patients about the benefits of EPC. Personalized symptom management according to ESAS score, nutritional assessment, coping and holistic support for patients and caregiver, the extent of awareness of diagnosis and prognosis, guidance in decision‐making and future planning, are specific elements of SCOC consultation. Patients keep on receiving cancer‐directed treatment, and through advance care planning receive specific care from the other specialists based on the identified needs. Annually, about 220 outpatients are managed through SCOC consultation.

Satisfaction has been associated with better patient‐physician relationship and, in patients with advanced cancer, with improved emotional functioning, global health, and quality of life.^10^ Satisfaction with care, which is highly relevant to patient‐centered care and quality improvement in healthcare, has not commonly been studied as an outcome in EPC trials.^11^ A few trials in EPC evaluated patient satisfaction outcomes, with some studies reporting significant improvement, while the type of model used to provide EPC did not impact study results.^12^ The FAMECARE‐P instrument of 13‐item measure of outpatients' satisfaction in EPC setting has been developed and validated in 2009 by Princess Margaret Hospital of Toronto, Canada.^13^ The FAMECARE‐P questionnaire is mainly oriented toward symptom control and side effect, and is proposed as a tool to be repeated and to evaluate the benefits obtained from EPCs over time.

In our study, the primary objective is to verify whether the care relationship established in the SCOC was satisfactory for the patient, and assess the impact of such intervention of systematic early integration of palliative care on patient's satisfaction. The secondary objective is to evaluate if there are differences in patient satisfaction based on age, gender and type of cancer, in a robust patient's sample.

In order to assess patients' care experience during the SCOC consultation, an ad‐hoc predominatly quantitative questionnaire has been developed by the interdisciplinary SCOC team with 8‐item measure of patient satisfaction. In particular, the questionnaire explores patients' feeling about time dedicated, being listened to, being understood, being able to speak openly; utility of consultation; information and indication received; evaluate the level of empathy of health care and quality of the relationship perceived; identify the perception of level of professional/quality of performance.

Herewith we report the results of this study that involved a robust sample of consecutive subjects affected by advanced cancer assessed within the SCOC, with the aim of exploring their perception of quality of this service.

## 2 MATERIALS AND METHODS

This was a cross‐sectional study in outpatients with advanced cancer (metastatic or locally advanced) admitted at SCOC consultation. The study was conducted between May 4 and December 7, 2022, at Medical Oncology Unit 1, IOV, Comprehensive Cancer Centre in Padua, Italy. Patients provided written informed consent, and the study was approved by the Ethics Committee of the IOV. An ad‐hoc questionnaire has been developed by SCOC interdisciplinary team. The items in the questionnaire were proposed by the oncologist and the psychologist of the team and then shared with the other members of the team (palliative care physician, clinical nutrition specialist and a nurse navigator). The questionnaire included demographic characteristics, tumor type and stage, and through a Likert scale it explored eight aspects of the consultation, in particular: time dedicated, feel listened to, feel understood, feel free to speak openly and to express doubts and concerns, utility of consultation, feeling about information and indication received, level of empathy of health care and quality of the relationship, and level of professional/quality of performance. Furthermore, the patient was asked how they experienced the presence of several professionals at the same time, and finally, an open question that collects comments and suggestions about SCOC was delivered (See questionnaire in Table S1). We then assessed eventual differences in questionnaire response according to age (< or 70 years old or more), gender and type of cancer.

All 174 consecutive patients with advanced cancer visited between May 4 and December 7, 2022 at SCOC, have been recruited for the study. Criteria for inclusion were: age ≥18 years and ability to read, understand and fill in the proposed questionnaire. Exclusion criteria were: presence of mental or psychiatric pathological conditions interfering with the state of consciousness or the ability to judge, patient's refusal to participate in the study.

The aim of the survey and the study were described and presented to patients at the end of SCOC consultation. Patients were asked to complete anonymously a self‐administered questionnaire. The questionnaire was in written form on paper and it was filled in by the patient after the SCOC consultation. The patient was not helped in the compilation in order not to create bias. The questionnaire, being anonymous, didn't require the patient names, but asked for some general information, such as: gender, age and diagnosis. The record of the databases was collected by person outside the clinic, so as to ensure the complete anonymity. Participation was voluntary.

### 2.1 Statistical analysis

Patients' characteristics and patients' perception of the SCOC consultation were described with descriptive analysis. Differences in the distribution of the questionnaire's variables with Likert scale were evaluated by gender, age and type of tumor. The comparisons were performed by Fisher's exact test.

Bar‐plot was used to summarize the questionnaire's variables with Likert scale.

Statistical analyses were performed by R Version 4.3.1. The level of significance was set at 5%.

## 3 RESULTS

The study was proposed to 174 patients, and 162 (93.1%) accepted to take part. The exclusion of these 12 (6.9%) patients was due to: refusal or inability to fill in the questionnaire. Patients' characteristics are reported in Table 1. Males were 108 (66.7%), median age was 71 years (range 62–77), with 91 patients (56.2%) aged 70 years or older. Patients were most often carrying a diagnosis of gastrointestinal cancer (119 patients, 73.5%) and a metastatic disease (143 patients, 88.3%), only 19 patients (11.7%) were locally advanced.

**TABLE 1:** Patients' characteristics.

Variables	*n* (%) 162 (100)
Gender	
Male	108 (66.7)
Female	54 (33.3)
Median age (IQR)	71 (62–77)
<70 years	71 (43.8)
≥70 years	91 (56.2)
Tumor site	
Gastrointestinal	119 (73.5)
Other tumor	43 (26.5)
Tumor stage	
Locally advanced	19 (11.7)
Metastatic	143 (88.3)

**FIGURE 1:**
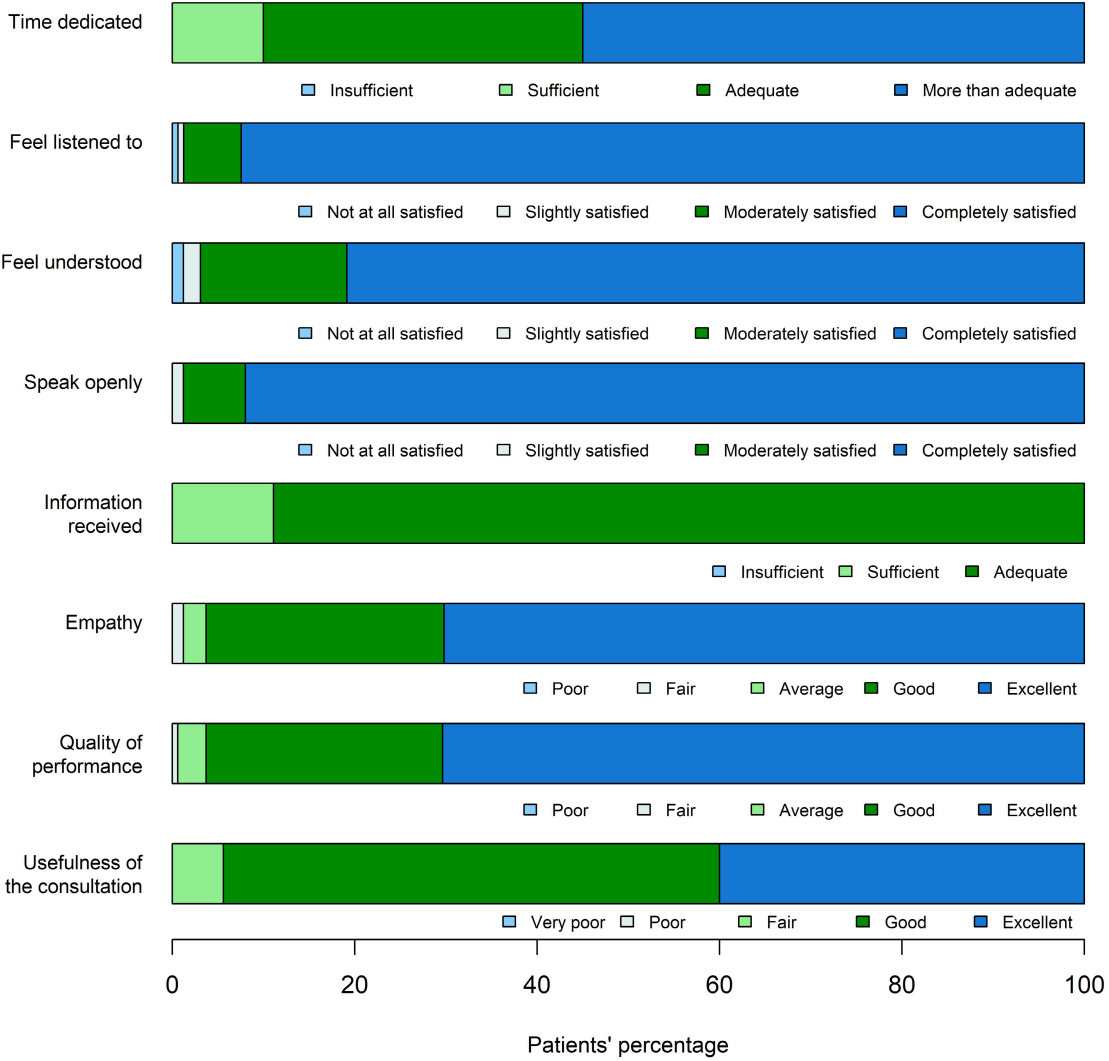
Patient satisfaction in the eight areas queried.

Answers to the questionnaire were collected and we identified patterns of quality associated with SCOC consultation. Figure 1 describes eight patient satisfaction areas and results obtained. In particular, half of the patients (55%) thought the time dedicated was more than adequate and 56 (35%) patients judged it adequate.

Regarding patient's perception about healthcare personnel “making room” and attention received, 147 (92.5%) patients were completely satisfied with being listened to, 131 (80.9%) patients were also completely satisfied with understanding of their problems and 149 (92%) patients felt free to speak openly and express doubts and concerns. Two subjects (1.2%) were not satisfied with regard to understanding of their problems and one (0.6%) with being listened to.

With regard to information and indications received, a maximum rating (“adequate”) was expressed by 144 (88.9%) patients, while the other patients reporting it as sufficient (18 patients, 11.1%). One hundred and thirteen patients (70.2%) evaluated the level of empathy and the quality of the relationship as excellent, while they were considered good by 42 (26.1%) patients. The level of professionalism and quality of performance were judged excellent by 114 (70.4%) patients and good by 42 (25.9%) patients. None of the questions received a poor rating.

The utility of SCOC consultation was considered excellent by 64 (40%) patients, good by 87 (54.4%) patients and fair by 9 (5.6%) patients. Both the questions had no negative feedback.

With regard to the presence of multiple professionals at SCOC consultation, eighty‐nine patients (56%) felt safe, 71 (44.6%) felt satisfied, 6 (3.8%) were surprised and two (1.3%) patients felt uncomfortable. No patient felt embarrassed.

No statistically significant differences were found in the responses to the questionnaire questions, by gender, age and type of tumor; except for question on the presence of more professionals at SCOC consultation. In this question there was a difference between the responses of elderly patients and those of adult patients, where “satisfied” was predominant compared to “safe” (53.8% vs 31% in adults, *p* = 0.0032) (See Table S2).

Regarding the qualitative part of the questionnaire, Table 2 reports the outcome of the open question. Thirty‐one patients (19.1%) formulated comments. All patients' comments and suggestions were positive with 11 (35.5%) expressing gratitude for the interdisciplinary evaluation and 11 (35.5%) a positive opinion of the team. Seven patients (22.6%) reported satisfaction with the SCOC consultation which responded to their needs. Interestingly, two patients suggested offering this interdisciplinary approach earlier in the care pathway.

**TABLE 2:** Open question: comments and suggestions.

Type	*n* (%) 31 (19.1)
Gratitude	11 (35.5)
Positive opinion of the team	11 (35.5)
Satisfaction with the consultation that responded to their needs	7 (22.6)
Suggest such consultation were done more often and/or more earlier	2 (6.4)

## 4 DISCUSSION

SCOC is a consolidated approach at IOV aiming at improving quality of care for patients with advanced‐stage cancer and their family. The fully embedded model activated in the Oncology Department, in which palliative care team and oncologist share the SCOC, meets all the criteria suggested by the international consensus to ensure timely activation of palliative care,^14^ as well as the best level of full integration.^2^ Measuring performance in service delivery as well as quantifying the level of integration is of utmost importance for a process of continuous improvement of services.^15^ A useful method to assess service quality is measuring the levels of satisfaction that users and frontline service providers ascribe to that service.^10^, ^11^, ^16^ Patients who participated in this study are essential stakeholders for the Oncology Department, and their opinions are crucial in addressing SCOC perception. Measuring patients' satisfaction is necessary to improve the provision of SCOC.^17^ Involving patients in the organization reframing of services can lead to authentic personalized medicine, and help healthcare providers making choices which are both informed and consistent with patients' needs.^18^

Patients with advanced cancer have a high burden of unmet needs, with special regard to symptoms control, psychological and emotional support and communication.^19^, ^20^ There is evidence showing that dissatisfaction with medical information and communication is associated with anxiety and depression which, in turn, contribute to the global burden of disability in patients with advanced cancer.^21^ Indeed, these issues should be addressed as priorities in order to improve patients' care.^20^ As reported by Zimmermann, EPC intervention is not a static process, and it requires verification and implementation through qualitative research.^7^ In 2004, a retrospective study by Strasser and colleagues on 138 consecutive patients with advanced cancer referred to palliative care team (stand‐alone clinics) evaluated symptoms' assessment and patient satisfaction of such approach.^22^ Beyond physician and a nurse evaluation, patients were assessed by a nutritionist, a pharmacist, a social worker, physical, occupational, and speech therapist, a psychiatric nurse practitioner, and a pastoral care worker. This palliative care clinic model, implemented at MD Anderson Cancer Centre,^23^ with half‐day symptom control, was shown to be associated with a reduction of the physical and psychological distress of patients. In particular, 83 patients of the service (60%) answering the questionnaire reported high level of satisfaction for the care team members (97%), adequate for assessment and treatment plan (91%), and “overall felt I was helped” (94%).^22^ The FAMCARE‐Patient scale measuring satisfaction with care over time in outpatients with advanced cancer, demonstrated that patient satisfaction was correlated with communication and relationship with health care provides, with physical distress, and with caregiver satisfactions.^13^ This questionnaire provides for the first time a patient satisfaction measurement tool in EPC setting in a quite different model from IOV one.^24^ No other instruments developed for evaluating patient satisfaction in the EPC setting are available in the literature. More recently a randomized study has confirmed that a model of early and systematic integration of palliative and oncological care increases quality of life of patients with advanced cancer, measured by EORTC tests.^25^ Symptom control of common cancer symptoms, maintaining physical function and daily activities, and psychosocial care (e.g., depression and distress), were perceived to be important for providing high‐quality cancer care, as reported by a recently review on 34 studies.^26^

In our cross‐sectional study which involved a robust sample of consecutive subjects we analyzed patients' perception and satisfaction of the embedded model at SCOC, with instrument quite similar to FAMCARE‐P scale. During the consultation, team spent time with patients and their families/caregivers to learn about their life experience: they discussed patient's specific context for treatment planning; assessed pain and other symptoms, provided insights on the trajectory of the illness; acknowledged cultural beliefs and values; developed a patient‐centered plan of action; paying special attention to the multiple and complex aspects of awareness of cancer prognosis and human dignity.^8^, ^9^

The results of our study demonstrate a high level of patients' satisfaction with SCOC consultation in consecutive patients corresponding to 74% of patients taken care of at SCOC in a year: more than 92% declare that they are completely satisfied with a feeling of being listened to, for the freedom to speak openly and express their doubts; 88.9% of patients consider the level of information received as excellent; 80.9% of patients felt completely understood for their difficulties. Moreover, the vast majority had an overall positive feedback for the level of empathy demonstrated by clinicians (excellent for 70.2% of patients and good for 26.1%) as well as for health professionals' competence (excellent for 70.4% and good for 25.9% of patients). In addition, time dedicated to the visit was judged either adequate or more than adequate by the vast majority of patients, with an overall usefulness of the SCOC consultation which was rated as high or very high by 94.4% of the subjects. The high approval rating was seen in all subgroups of patients (gender, age, and type of tumor).

As regards the open question, which explores the qualitative aspect of the questionnaire, all the comments were positive and usually accompanied by a deep sense of gratitude. Two patients suggested making this interdisciplinary approach available even in the earliest stages of the disease.

Our study findings extend the existing literature on benefits of EPC into the cancer patients journey,^3^, ^10^, ^11^, ^12^, ^13^, ^27^ which is also able to increase the satisfaction with care of their caregivers.^28^ Provided the importance of EPC has been widely reported, this study showed that a fully integrated model of SCOC (“embedded model”) is helpful to deliver care which enhances satisfaction from the patient's perspective. Satisfaction with care is a hallmark of healthcare quality,^10^, ^29^, ^30^ and the domains of satisfaction with care turned out with a high performance in every items explored by our questionnaire. Indeed, feeling supported by healthcare providers and having a trustful relationship with them in terms of good communication with the opportunity of sharing doubts and fears that may be contained are elements that promote the experience of hope as shown both in the context of end‐of‐life care^10^, ^31^ as well as during active oncological treatment.^32^ Patients' hope indeed has been demonstrated to be sustained by trusting relationships with their healthcare teams.^33^

Hoff and Cholette suggest that interdisciplinary setting request constant work on quality of communication within the team, degree of emotional cohesion and on the use of a shared mental model of care.^34^, ^35^ In our team, these aims are achieved through permanent training and limiting the turnover of personnel involved in SCOC.

Early referral to the palliative care team with the opportunity to build a relationship from the time of diagnosis of advanced cancer was shown beneficial for many patients.^28^ Most patients felt that within the palliative care clinic they could openly discuss end‐of‐life issues.^28^ Oncologists are the first line of providers for patients with advanced‐stage cancer; therefore, they should recognize and outline all of their needs in order to implement symptom control as well as a personal psychological and spiritual intervention, and to proper address patients at SCOC for a global take of care. Addressing coping strategies is associated with quality of life and depression improvements.^36^

Time dedicated to identifying patients' understanding of their illness, treatment and prognosis was a consistent factor for success in EPC setting.^37^

Honest communication discussing choices and outcomes is a mainstay to help patients effectively cope with their illness, and SCOC guidance allows patients to express their priorities and wishes particularly during the final steps of life.^38^ If the patient perceives the availability of time and listening of the healthcare team, he is more inclined to realistic hope and peacefully face end of life choices (place of death, palliative sedation, etc.). Members of the SCOC can help patients to boost their level of hope for the future in advanced cancer.

Finally, a widespread sense of gratitude, in some cases also restated in the open comments, emerged by results of this study, suggesting an emotional state related to the model of care, as suggested in other experiences in EPC deliver.^39^ This study encouraged us to enhance our practice of SCOC consultation.

These results suggest that our patients and oncologist, as reported in the literature, favored the embedded model on EPC deliver.^40^, ^41^

### 4.1 Strengths, limitations, and future research

This study has some limitations. Primarily, this is a single‐center study in a specific population; so, although the sample is quite large and highly representative of whole population annually assessed at SCOC (even for age, gender and kind of cancer), the results might not be extrapolated to other populations or clinics. Most importantly, it involves a peculiar embedded model of EPC consultation put in place in a tertiary cancer center, and therefore, it adds a piece to lack of knowledge about patient satisfaction related to EPC delivery models. The key components of SCOC team that proposed the questionnaire to the patients has been involved to develope, tailor and enhance integrated models for EPC for cancer patients, but at this time the role of each team members on the degree of patient satisfaction remains unclear. Future studies in this area are needed, particularly to determine whether and how the presence of the oncologist within the interdisciplinary palliative care team can facilitate communication, awareness, and shared choice with patients and family of the end of life for cancer patients. At the same time, other studies will be needed to understand whether the oncologist can provide the palliative care team with useful information to better frame the prognosis and patients' expectations.

## 5 CONCLUSIONS

Our study provides evidence favoring ECP in embedded model, with regard to satisfaction with care, and the patient‐clinician relationship is confirmed to be paramount throughout illness as well as a central determinant of patients' perceptions of quality of care.^11^ The results of our study showed a high overall level of patient satisfaction, that has proven to be independent of age, gender and type of tumor. Feedback from patients proved the model put in place to be effective in helping them shape their treatment journey and decision at the end of life. It also highlighted areas where we could have been even more effective in improving our ability to listen and communicate.^39^ We can conclude that SCOC has achieved its goal of offering holistic support to advanced cancer patients and family members.

Finally, we are reminded that it is vital to budget enough time to reflect on patients' care experience so as to remain effectively patient centered,^42^, ^43^ even in earlier phases of disease, as suggested by some patients.

## AUTHOR CONTRIBUTIONS

**Antonella Galiano:** Conceptualization (equal); data curation (equal); investigation (equal); project administration (equal); visualization (equal); writing – original draft (equal); writing – review and editing (equal). **Alessandra Feltrin:** Conceptualization (equal); data curation (equal); investigation (equal); writing – review and editing (equal). **Ardi Pambuku:** Investigation (equal); writing – review and editing (equal). **Leda Lo Mauro:** Investigation (equal); writing – review and editing (equal). **Chiara De Toni:** Data curation (equal); formal analysis (equal); visualization (equal); writing – original draft (equal); writing – review and editing (equal). **Sabina Murgioni:** Investigation (equal); writing – review and editing (equal). **Caterina Soldà:** Investigation (equal); writing – review and editing (equal). **Marco Maruzzo:** Investigation (equal); writing – review and editing (equal). **Francesca Bergamo:** Investigation (equal); writing – review and editing (equal). **Antonella Brunello:** Data curation (equal); investigation (equal); visualization (equal); writing – original draft (equal); writing – review and editing (equal). **Vittorina Zagonel:** Conceptualization (equal); data curation (equal); investigation (equal); supervision (equal); visualization (equal); writing – original draft (equal); writing – review and editing (equal).

## FUNDING INFORMATION

This study was supported by Ricerca Corrente 2023, Italian Ministry of Health.

## CONFLICT OF INTEREST STATEMENT

Authors declare no competing interest.

## ETHICS STATEMENT

The study was conducted in accordance with the Declaration of Helsinki and was approved by the Veneto Institute of Oncology Ethics Committee (d.D.G. n. 1124/2022).

## PATIENTS CONSENT STATEMENT

Participants signed an informed consent for personal and health information data processing for scientific research.

## Supporting information


Table S1.

Table S2.

## Data Availability

Data presented in this study are available on request from the corresponding author.
