# Supplementary material for: What do cancer patients experience of the simultaneous care clinic? Results of a cross‐sectional study on patient care satisfaction
Source: Cancer Med. 2024 Feb 24;13(3):e7000. doi: 10.1002/cam4.7000 (PMC10891442; doi:10.1002/cam4.7000)
Supplement: Supplementary file 1 — Table S1. [file CAM4-13-e7000-s001.docx]

**Supplemental Online Table 1. Questionnaire.**

| 1. The time dedicated to you was: | | | | | | | | | | | | | |
| --- | --- | --- | --- | --- | --- | --- | --- | --- | --- | --- | --- | --- | --- |
| Insufficient | | | Sufficient | | | | Adequate | | | | More than adequate | | |
| 2. The presence of several doctors has been the reason for: | | | | | | | | | | | | | |
| Surprise | Embarrassment | | | | Discomfort | | Satisfaction | | | Safety | | | Other |
| 3. Did you feel listened to with regard to your problems? | | | | | | | | | | | | | |
| Not at all satisfied | | | Slightly satisfied | | | | Moderately satisfied | | | | Completely satisfied | | |
| 4. Did you feel understood with regard to your difficulties? | | | | | | | | | | | | | |
| Not at all satisfied | | | Slightly satisfied | | | | Moderately satisfied | | | | Completely satisfied | | |
| 5. Did you feel free to speak openly and to express doubts and concerns? | | | | | | | | | | | | | |
| Not at all satisfied | | | Slightly satisfied | | | | Moderately satisfied | | | | Completely satisfied | | |
| 6. What is your overall assessment of the information and indications received? | | | | | | | | | | | | | |
| Insufficient | | | | Sufficient | | | | | Adequate | | | | |
| 7. How do you rate the level of "empathy" of health care / quality of the relationship? | | | | | | | | | | | | | |
| Poor | | Fair | | | | Average | | Good | | | | Excellent | |
| 8. How do you rate the level of professionalism / quality of performance? | | | | | | | | | | | | | |
| Poor | | Fair | | | | Average | | Good | | | | Excellent | |
| 9. Thinking about your treatment path, did this consultation seem useful to you? | | | | | | | | | | | | | |
| Very poor | | Poor | | | | Fair | | Good | | | | Excellent | |
| Comments and suggestions | | | | | | | | | | | | | |
