# Supplementary material for: What do cancer patients experience of the simultaneous care clinic? Results of a cross‐sectional study on patient care satisfaction
Source: Cancer Med. 2024 Feb 24;13(3):e7000. doi: 10.1002/cam4.7000 (PMC10891442; doi:10.1002/cam4.7000)
Supplement: Supplementary file 2 — Table S2. [file CAM4-13-e7000-s002.docx]

**Supplemental Online Table 2. Distribution of questionnaire responses for gender, age and type of tumour.**

| **Questionnaire** | **Total**  162 (100) | **Female**  54 (33.3) | **Male**  108 (66.7) | **p-value** |
| --- | --- | --- | --- | --- |
| **1. The time dedicated to you was:** |  |  |  |  |
| Insufficient | 0 (0.0) | 0 (0.0) | 0 (0.0) | 0.4823 |
| Sufficient | 16 (10.0) | 7 (13.0) | 9 (8.5) |  |
| Adequate | 56 (35.0) | 16 (29.6) | 40 (37.7) |  |
| More than adequate | 88 (55.0) | 31 (57.4) | 57 (53.8) |  |
| **2. The presence of several doctors has been the reason for:** |  |  |  |  |
| Surprise | 6 (3.8) | 2 (3.7) | 4 (3.7) | 0.7470 |
| Embarrassment | 0 (0.0) | 0 (0.0) | 0 (0.0) |  |
| Discomfort | 2 (1.3) | 1 (1.9) | 1 (0.9) |  |
| Satisfaction | 71 (44.6) | 21 (38.9) | 50 (46.3) |  |
| Safety | 89 (56.0) | 33 (61.1) | 56 (51.8) |  |
| Other | 0 (0.0) | 0 (0.0) | 0 (0.0) |  |
| **3. Did you feel listened to with regard to your problems?** |  |  |  |  |
| Not at all satisfied | 1 (0.6) | 0 (0.0) | 1 (0.9) | 1 |
| Slightly satisfied | 1 (0.6) | 0 (0.0) | 1 (0.9) |  |
| Moderately satisfied | 10 (6.3) | 3 (5.7) | 7 (6.7) |  |
| Completely satisfied | 147 (92.5) | 50 (94.3) | 97 (91.5) |  |
| **4. Did you feel understood with regard to your difficulties?** |  |  |  |  |
| Not at all satisfied | 2 (1.2) | 0 (0.0) | 2 (1.9) | 0.2755 |
| Slightly satisfied | 3 (1.8) | 2 (3.7) | 1 (0.9) |  |
| Moderately satisfied | 26 (16.1) | 6 (11.1) | 20 (18.5) |  |
| Completely satisfied | 131 (80.9) | 46 (85.2) | 85 (78.7) |  |
| **5. Did you feel free to speak openly and to express doubts and concerns?** |  |  |  |  |
| Not at all satisfied | 0 (0.0) | 0 (0.0) | 0 (0.0) | 0.2725 |
| Slightly satisfied | 2 (1.2) | 0 (0.0) | 2 (1.9) |  |
| Moderately satisfied | 11 (6.8) | 6 (11.1) | 5 (4.6) |  |
| Completely satisfied | 149 (92.0) | 48 (88.9) | 101 (93.5) |  |
| **6. What is your overall assessment of the information and indications received?** |  |  |  |  |
| Insufficient | 0 (0.0) | 0 (0.0) | 0 (0.0) | 0.6037 |
| Sufficient | 18 (11.1) | 7 (13.0) | 11 (10.2) |  |
| Adequate | 144 (88.9) | 47 (87.0) | 97 (89.8) |  |
| **7. How do you rate the level of "empathy" of health care / quality of the relationship?** |  |  |  |  |
| Poor | 0 (0.0) | 0 (0.0) | 0 (0.0) | 0.6348 |
| Fair | 2 (1.2) | 0 (0.0) | 2 (1.9) |  |
| Average | 4 (2.5) | 1 (1.9) | 3 (2.8) |  |
| Good | 42 (26.1) | 17 (31.5) | 25 (23.3) |  |
| Excellent | 113 (70.2) | 36 (66.6) | 77 (72.0) |  |
| **8. How do you rate the level of professionalism / quality of performance?** |  |  |  |  |
| Poor | 0 (0.0) | 0 (0.0) | 0 (0.0) | 0.8084 |
| Fair | 1 (0.6) | 0 (0.0) | 1 (0.9) |  |
| Average | 5 (3.1) | 1 (1.9) | 4 (3.7) |  |
| Good | 42 (25.9) | 16 (29.6) | 26 (24.1) |  |
| Excellent | 114 (70.4) | 37 (68.5) | 77 (71.3) |  |
| **9. Thinking about your treatment path, did this consultation seem useful to you?** |  |  |  |  |
| Very poor | 0 (0.0) | 0 (0.0) | 0 (0.0) | 0.3449 |
| Poor | 0 (0.0) | 0 (0.0) | 0 (0.0) |  |
| Fair | 9 (5.6) | 3 (5.6) | 6 (5.6) |  |
| Good | 87 (54.4) | 33 (62.3) | 54 (50.5) |  |
| Excellent | 64 (40.0) | 17 (32.1) | 47 (43.9) |  |
| **Questionnaire** | **Total**  162 (100) | **<70 years**  71 (43.8) | **≥70 years**  91 (56.2) | **p-value** |
| **1. The time dedicated to you was:** |  |  |  |  |
| Insufficient | 0 (0.0) | 0 (0.0) | 0 (0.0) | 0.1665 |
| Sufficient | 16 (10.0) | 4 (5.6) | 12 (13.5) |  |
| Adequate | 56 (35.0) | 29 (40.9) | 27 (30.3) |  |
| More than adequate | 88 (55.0) | 38 (53.5) | 50 (56.2) |  |
| **2. The presence of several doctors has been the reason for:** |  |  |  |  |
| Surprise | 6 (3.8) | 5 (7.0) | 1 (1.1) | 0.0032 |
| Embarrassment | 0 (0.0) | 0 (0.0) | 0 (0.0) |  |
| Discomfort | 2 (1.3) | 2 (2.8) | 0 (0.0) |  |
| Satisfaction | 71 (44.6) | 22 (31.0) | 49 (53.8) |  |
| Safety | 89 (56.0) | 47 (66.2) | 42 (46.1) |  |
| Other | 0 (0.0) | 0 (0.0) | 0 (0.0) |  |
| **3. Did you feel listened to with regard to your problems?** |  |  |  |  |
| Not at all satisfied | 1 (0.6) | 1 (1.4) | 0 (0.0) | 0.4608 |
| Slightly satisfied | 1 (0.6) | 1 (1.4) | 0 (0.0) |  |
| Moderately satisfied | 10 (6.3) | 5 (7.1) | 5 (5.7) |  |
| Completely satisfied | 147 (92.5) | 64 (90.1) | 83 (94.3) |  |
| **4. Did you feel understood with regard to your difficulties?** |  |  |  |  |
| Not at all satisfied | 2 (1.2) | 1 (1.4) | 1 (1.1) | 0.6551 |
| Slightly satisfied | 3 (1.8) | 2 (2.8) | 1 (1.1) |  |
| Moderately satisfied | 26 (16.1) | 9 (12.7) | 17 (18.7) |  |
| Completely satisfied | 131 (80.9) | 59 (83.1) | 72 (79.1) |  |
| **5. Did you feel free to speak openly and to express doubts and concerns?** |  |  |  |  |
| Not at all satisfied | 0 (0.0) | 0 (0.0) | 0 (0.0) | 0.4057 |
| Slightly satisfied | 2 (1.2) | 1 (1.4) | 1 (1.1) |  |
| Moderately satisfied | 11 (6.8) | 7 (9.9) | 4 (4.4) |  |
| Completely satisfied | 149 (92.0) | 63 (88.7) | 86 (94.5) |  |
| **6. What is your overall assessment of the information and indications received?** |  |  |  |  |
| Insufficient | 0 (0.0) | 0 (0.0) | 0 (0.0) | 1 |
| Sufficient | 18 (11.1) | 8 (11.3) | 10 (11.0) |  |
| Adequate | 144 (88.9) | 63 (88.7) | 81 (89.0) |  |
| **7. How do you rate the level of "empathy" of health care / quality of the relationship?** |  |  |  |  |
| Poor | 0 (0.0) | 0 (0.0) | 0 (0.0) | 0.1101 |
| Fair | 2 (1.2) | 1 (1.4) | 1 (1.1) |  |
| Average | 4 (2.5) | 3 (4.2) | 1 (1.1) |  |
| Good | 42 (26.1) | 13 (18.3) | 29 (32.2) |  |
| Excellent | 113 (70.2) | 54 (76.1) | 59 (65.6) |  |
| **8. How do you rate the level of professionalism / quality of performance?** |  |  |  |  |
| Poor | 0 (0.0) | 0 (0.0) | 0 (0.0) | 0.1294 |
| Fair | 1 (0.6) | 0 (0.0) | 1 (1.1) |  |
| Average | 5 (3.1) | 3 (4.2) | 2 (2.2) |  |
| Good | 42 (25.9) | 13 (18.3) | 29 (31.9) |  |
| Excellent | 114 (70.4) | 55 (77.5) | 59 (64.8) |  |
| **9. Thinking about your treatment path, did this consultation seem useful to you?** |  |  |  |  |
| Very poor | 0 (0.0) | 0 (0.0) | 0 (0.0) | 0.2363 |
| Poor | 0 (0.0) | 0 (0.0) | 0 (0.0) |  |
| Fair | 9 (5.6) | 2 (2.9) | 7 (7.8) |  |
| Good | 87 (54.4) | 36 (51.4) | 51 (56.7) |  |
| Excellent | 64 (40.0) | 32 (45.7) | 32 (35.5) |  |
| **Questionnaire** | **Total**  162 (100) | **Other**  43 (26.5) | **GI**  119 (73.5) | **p-value** |
| **1. The time dedicated to you was:** |  |  |  |  |
| Insufficient | 0 (0.0) | 0 (0.0) | 0 (0.0) | 0.7218 |
| Sufficient | 16 (10.0) | 5 (11.9) | 11 (9.3) |  |
| Adequate | 56 (35.0) | 13 (31.0) | 43 (36.5) |  |
| More than adequate | 88 (55.0) | 24 (57.1) | 64 (54.2) |  |
| **2. The presence of several doctors has been the reason for:** |  |  |  |  |
| Surprise | 6 (3.8) | 0 (0.0) | 6 (5.0) | 0.3847 |
| Embarrassment | 0 (0.0) | 0 (0.0) | 0 (0.0) |  |
| Discomfort | 2 (1.3) | 0 (0.0) | 2 (1.7) |  |
| Satisfaction | 71 (44.6) | 18 (41.9) | 53 (44.5) |  |
| Safety | 89 (56.0) | 25 (58.1) | 64 (53.8) |  |
| Other | 0 (0.0) | 0 (0.0) | 0 (0.0) |  |
| **3. Did you feel listened to with regard to your problems?** |  |  |  |  |
| Not at all satisfied | 1 (0.6) | 0 (0.0) | 1 (0.8) | 0.8411 |
| Slightly satisfied | 1 (0.6) | 0 (0.0) | 0 (0.8) |  |
| Moderately satisfied | 10 (6.3) | 3 (7.5) | 7 (5.9) |  |
| Completely satisfied | 147 (92.5) | 37 (92.5) | 110 (92.5) |  |
| **4. Did you feel understood with regard to your difficulties?** |  |  |  |  |
| Not at all satisfied | 2 (1.2) | 1 (2.3) | 1 (0.8) | 0.4392 |
| Slightly satisfied | 3 (1.8) | 0 (0.0) | 3 (2.5) |  |
| Moderately satisfied | 26 (16.1) | 9 (20.9) | 17 (14.3) |  |
| Completely satisfied | 131 (80.9) | 33 (76.8) | 98 (82.4) |  |
| **5. Did you feel free to speak openly and to express doubts and concerns?** |  |  |  |  |
| Not at all satisfied | 0 (0.0) | 0 (0.0) | 0 (0.00) | 0.2210 |
| Slightly satisfied | 2 (1.2) | 1 (2.3) | 1 (0.8) |  |
| Moderately satisfied | 11 (6.8) | 1 (2.3) | 10 (8.4) |  |
| Completely satisfied | 149 (92.0) | 41 (95.4) | 108 (90.8) |  |
| **6. What is your overall assessment of the information and indications received?** |  |  |  |  |
| Insufficient | 0 (0.0) | 0 (0.0) | 0 (0.0) | 0.2571 |
| Sufficient | 18 (11.1) | 7 (16.3) | 11 (9.2) |  |
| Adequate | 144 (88.9) | 36 (83.7) | 108 (90.8) |  |
| **7. How do you rate the level of "empathy" of health care / quality of the relationship?** |  |  |  |  |
| Poor | 0 (0.0) | 0 (0.0) | 0 (0.00) | 0.8673 |
| Fair | 2 (1.2) | 1 (2.3) | 1 (0.9) |  |
| Average | 4 (2.5) | 1 (2.3) | 3 (2.5) |  |
| Good | 42 (26.1) | 11 (25.6) | 31 (26.3) |  |
| Excellent | 113 (70.2) | 30 (69.8) | 83 (70.3) |  |
| **8. How do you rate the level of professionalism / quality of performance?** |  |  |  |  |
| Poor | 0 (0.0) | 0 (0.0) | 0 (0.0) | 0.4755 |
| Fair | 1 (0.6) | 1 (2.3) | 0 (0.0) |  |
| Average | 5 (3.1) | 1 (2.3) | 4 (3.4) |  |
| Good | 42 (25.9) | 10 (23.3) | 32 (29.9) |  |
| Excellent | 114 (70.4) | 31 (72.1) | 83 (69.7) |  |
| **9. Thinking about your treatment path, did this consultation seem useful to you?** |  |  |  |  |
| Very poor | 0 (0.0) | 0 (0.0) | 0 (0.0) | 0.7640 |
| Poor | 0 (0.0) | 0 (0.0) | 0 (0.0) |  |
| Fair | 9 (5.6) | 2 (4.8) | 7 (5.9) |  |
| Good | 87 (54.4) | 21 (50.0) | 66 (55.9) |  |
| Excellent | 64 (40.0) | 19 (45.2) | 45 (38.2) |  |
